# The lactylation-macrophage interplay: implications for gastrointestinal disease therapeutics

**DOI:** 10.3389/fimmu.2025.1608115

**Published:** 2025-07-09

**Authors:** Xinzhen Che, Yixin Zhang, Xiqi Chen, Guangdong Xie, Jinling Li, Chengchao Xu, Chunhua Zhang, Yong Zhu, Xinyu Yang

**Affiliations:** ^1^ First Clinical Medical College, Shandong University of Traditional Chinese Medicine, Jinan, Shandong, China; ^2^ Affiliated Hospital of Shandong University of Traditional Chinese Medicine, Jinan, Shandong, China; ^3^ College of Acupuncture and Tuina, Shandong University of Traditional Chinese Medicine, Jinan, Shandong, China; ^4^ Rehabilitation Medicine College, Shandong University of Traditional Chinese Medicine, Jinan, Shandong, China; ^5^ Shandong Provincial Hospital Affiliated to Shandong First Medical University, Jinan, Shandong, China

**Keywords:** lactate, histone lactylation, macrophage plasticity, inflammatory bowel disease, postoperative ileus, gastrointestinal oncology

## Abstract

Lactate, a key metabolic byproduct of the Warburg effect, has lately been recognized as a regulator of histone lysine lactylation, a unique post-translational modification that plays a crucial role in essential biological processes, including the regulation of gene transcription. Lactylation plays a crucial regulatory role in macrophage biology by influencing inflammatory responses, tumor immune evasion, and fibrotic development. This review methodically investigates the molecular mechanisms of lactate metabolism and lactylation modification, focusing on their roles in macrophage activation and polarization in relation to gastrointestinal disorders, such as gastric cancer, colorectal carcinoma, ulcerative colitis, postoperative ileus, and bacterial and viral gastrointestinal infections. We clarify the molecular switching role of lactylation in regulating macrophage polarization under pathological settings by integrating current developments in epigenetic regulation and metabolic reprogramming. Current evidence demonstrates the dual regulatory role of lactylation in macrophage-mediated immune responses: it fosters anti-inflammatory and reparative phenotypes, yet may paradoxically expedite tumor progression and induce immunosuppressive conditions in certain gastrointestinal microenvironments. This review emphasizes that exploring lactylation as a novel therapeutic target offers new insights into gastrointestinal pathogenesis and lays a molecular groundwork for formulating precision therapeutic strategies against inflammatory diseases and malignant tumors.

## 1 Introduction

Gastrointestinal diseases include a range of medical problems, such as functional dyspepsia, celiac disease, inflammatory bowel disease (IBD), and gastrointestinal cancers (1). About half of the gastrointestinal symptoms seen in primary care are due to real medical issues, which are divided into two groups: organic disorders (like chronic atrophic gastritis, peptic ulcers, inflammatory bowel disease, and gastrointestinal cancers) and functional disorders (2–4). Data from the American Cancer Society indicates that by 2025, there will be roughly 30,300 new instances of gastric cancer and 154,270 new instances of colorectal cancer in the United States. We will classify these cancers as the third most prevalent kind, accounting for 42.6% of gastrointestinal tumors. The incidence rate among adults aged 50 and above is rising at an annual rate of 2.4% (5). In the United States, over 1% of the population, or 1 in every 100 individuals, is afflicted by inflammatory bowel disease (IBD), with ulcerative colitis (UC) and Crohn’s disease (CD) exhibiting similar incidence rates. The global prevalence of inflammatory bowel disease (IBD) positively corresponds with the level of industrialization, with developing nations witnessing an increasing incidence (6). The pathogenesis of these diseases involves multidimensional mechanisms, including pro-inflammatory cytokine cascades, gut microbiota dysbiosis, immune dysregulation, epithelial barrier repair dysfunction, and tumor microenvironment remodeling (7). Thus, clarifying the molecular interactions that drive these disease processes and creating precision therapies aimed at the microenvironment have become essential focuses in translational medicine research.

Lactate, functioning as a pivotal metabolic node in the glycolytic and mitochondrial oxidative phosphorylation homeostatic network, is dynamically regulated by the equilibrium between the Warburg effect and reverse Warburg effect (8). Groundbreaking studies have redefined lactate not merely as an end-product of hypoxic metabolism, but as a recyclable carbon source among multicellular populations within the tumor microenvironment (TME), while also serving as a metabolic signaling molecule that participates in immunometabolic regulation through epigenetic reprogramming (9, 9). Research has shown that under conditions of hypoxia, increased metabolic activity, and inflammation, anaerobic yeasts produce a large amount of lactate through glycolysis (10). Mechanistic investigations demonstrate that under hypoxic stress, hyperactive energy metabolism, and chronic inflammation, the glycolytic rate-limiting enzyme LDHA (lactate dehydrogenase A) undergoes significant activation, driving exponential lactate production (11). This pathological lactate accumulation bidirectionally modulates NF-κB-mediated inflammatory cascades and PD-L1-associated tumor immune evasion mechanisms via GPR81/AMPK signaling axis activation (12, 13). As a novel lactate-derived post-translational modification (PTM) (14). The molecular basis of lactylation was first elucidated by Zhang et al. (8) in 2019. Their seminal work demonstrated that lactate covalently modifies histone H3K18 sites (H3K18la), establishing an epigenetic regulatory interface that dictates macrophage polarization fate (M1/M2 switching) and immune checkpoint molecule expression profiles. Cutting-edge research reveals that histone lactylation remodels 3D chromatin architecture to specifically activate metabolic stress response networks (e.g., HIF-1α, mTORC1 pathways), while regulating key effectors involved in immune activation (CD8+ T cell infiltration), metabolic reprogramming (glutaminolysis), and tissue regeneration (VEGF signaling) (15).

**Figure 1:**
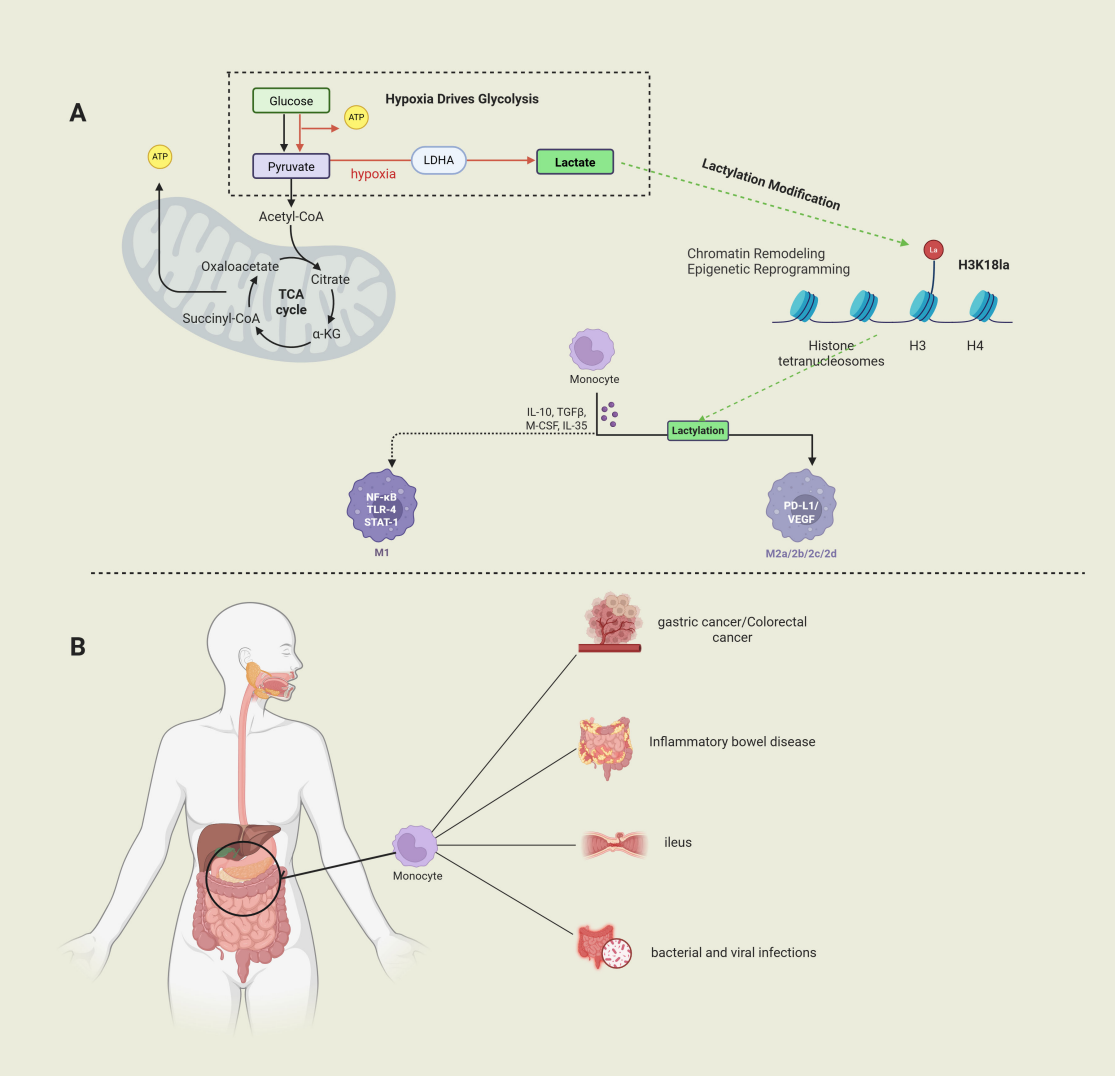
(Image 1 by Biorender) Part **(A)** Under hypoxic conditions, glucose generates pyruvate through glycolysis and is converted to lactate; lactate regulates histone function through lactation modifications (e.g., H3K18la) and regulates macrophage polarization. Part **(B)** Macrophages play a key role in diseases such as gastric cancer, colorectal cancer, inflammatory bowel disease, and bacterial/viral infections and may be involved in the pathological process by modulating the immune microenvironment.

Macrophages, as key members of the mononuclear phagocyte system, play a crucial role in immune defense, tissue homeostasis maintenance, and anti-tumor immune surveillance (16, 17). With their high plasticity, macrophages perform a variety of functions in both homeostasis and immune responses. Through polarization, macrophages carry out immune functions responsible for microbial defense, while also participating in the disease processes of autoimmune diseases and malignant tumors (18). Notably, in gastrointestinal pathologies, macrophage polarization exhibits dual regulatory characteristics—M1 polarization exacerbates mucosal damage via NF-κB/STAT3 signaling in inflammatory bowel disease (IBD) (19), whereas tumor-associated macrophage (TAM) M2 polarization promotes angiogenesis and immune evasion through VEGF/PD-L1 axis in gastric and colorectal cancer microenvironments (20, 21). The molecular switch mechanism governing macrophage transition from pro-inflammatory to reparative phenotypes remains incompletely elucidated. Emerging mechanistic studies reveal lactate’s multimodal regulation of macrophage activation: (1) as a HIF-1α stabilizer inducing M2 polarization;(2) via GPR81-mediated NLRP3 inflammasome suppression; (3) through histone H3K18 lactylation-mediated chromatin accessibility remodeling (22). These discoveries establish a theoretical foundation for developing novel therapeutic strategies targeting the lactate-macrophage axis, particularly highlighting its translational potential in rebalancing intestinal immune microenvironments and reversing tumor immunosuppression (**Figure 1**).

## 2 Lactylation: a metabolic-epigenetic crosstalk hub

Metabolites and intermediates not only play important roles in metabolic processes but also possess non-metabolic functions in cellular signal transduction. Lactate, traditionally viewed as an end-product of glycolysis, has been redefined as a multidimensional signaling molecule that regulates cell fate determination through epigenetic reprogramming (23). Lactylation, a post-translational modification (PTM) driven by intracellular lactate accumulation, is biochemically characterized by the covalent conjugation of a lactyl group to the ϵ-amino group of lysine residues on target proteins (24). In 2019, the Zhang team first reported the lactylation modification of histone H3K18 and elucidated its molecular mechanism in regulating macrophage polarization and inflammatory responses by modulating chromatin’s three-dimensional conformation (8, 25). The discovery of histone lactylation modification reveals lactate-dependent, dynamic, and reversible epigenetic changes that can regulate gene expression and precisely control cellular metabolism (26). Lactylation is currently considered to have two isomers, namely L-lactyl (K(L-la)) and D-lactyl (K(D-la)) configurations (8, 27).

Lactate (C_3_H_6_O_3_), the terminal product of glycolysis, is a hydroxycarboxylic acid generated through NADH-dependent reduction of pyruvate catalyzed by lactate dehydrogenase (LDH). Its stereochemical configurations include L- (levorotatory), D- (dextrorotatory), and racemic DL-forms, with bioactive L-lactate constituting over 95% of the mammalian lactate pool (14, 28, 29). Pyruvate, a pivotal glucose catabolism intermediate, resides at a metabolic branch point: mitochondrial conversion to acetyl-CoA via the pyruvate dehydrogenase complex (PDC) for oxidative phosphorylation, or cytosolic LDH-mediated reduction to lactate (14, 30). Lactate metabolism has dual pathways: Lactate metabolism bifurcates into: ① mitochondrial import via monocarboxylate transporters (MCTs) and reconversion to pyruvate by LDHB for TCA cycle entry; ② hepatic/renal gluconeogenesis (Cori cycle) (31).

The seminal Warburg effect in oncology reveals tumor cells’ preferential glycolysis despite oxygen sufficiency, yielding copious lactate with rapid ATP generation (32). Among them, LDHA is one of the key enzymes for glucose metabolism reprogramming in the TME. It promotes the conversion of pyruvate to lactate, and its activity is positively correlated with the Warburg effect (33), which directly or indirectly activates signal transduction pathways and modulates immune responses to be involved in tumorigenesis and progression (34). The Warburg effect explains the central role of lactate in tumor metabolism. As a novel epigenetic mechanism, lactate triggers histone lysine lactylation modifications, thereby controlling various biological processes such as tumor initiation, progression, immune evasion, and cancer cell metabolic reprogramming (35, 36). During inflammation, tissue repair requires a large amount of energy, with glycolysis being abnormally active to ensure that lactate concentrations inside and outside the cells are higher than those in resting-state cells. Meanwhile, lactate also serves as fuel for mitochondrial metabolism, providing the large amounts of energy required. In summary, lactate accumulation is an inevitable result of inflammatory diseases and tumors (36–38). Conversely, it can influence the occurrence and development of diseases by regulating immune responses and tumor immunity (39). In addition to the Warburg effect in cancer cells, it is also present in proliferating T cells, macrophages, and fibroblasts (40). Lactate homeostasis is maintained by the lactate shuttle system, comprising: ① Monocarboxylate Transporters (MCT) isoforms (MCT1 for uptake, MCT4 for efflux); ② Proton gradient-dependent cotransport (41, 42). Based on the lactate shuttle hypothesis, lactate is described as a carrier linking glycolysis and oxidative metabolism. The association between lactate in the glycolytic pathway and aerobic pathways can occur continuously under fully aerobic conditions. The lactate shuttle can overcome the cellular compartmentalization barrier (43). System dynamics are governed by spatiotemporal expression of LDH isozymes (LDHA favoring lactate production, LDHB promoting oxidation) and MCT subtypes (44).

Epigenetic modifications refer to biological processes that regulate gene expression through DNA sequence-independent mechanisms, characterized by spatiotemporal control of gene activity via chromatin structural remodeling (45). As a pivotal epigenetic regulatory modality, histone acylation exhibits reversibility, spatiotemporal dynamics, and evolutionary conservation, orchestrating embryogenesis, tissue differentiation, and cellular stress responses to maintain organismal homeostasis (46). These modifications are catalyzed by specific acyltransferases that covalently conjugate acyl groups to histone lysine residues, with major types including acetylation, methylation, phosphorylation, and the newly discovered lactylation (47).

The dynamic equilibrium of histone lactylation is co-regulated by “writers” (lactyltransferases) and “erasers” (delactylases), involving the covalent addition and removal of lactyl groups and chromatin structure remodeling. In eukaryotes, the chromatin core structural unit is the nucleosome—a disk-like structure formed by a histone octamer (two each of H2A, H2B, H3, H4) wrapped with 147bp DNA, organized into higher-order chromatin fibers via linker histone H1 (48, 49). Cutting-edge research has unveiled the competitive binding between lactylation and acetylation, shedding light on the metabolic regulatory dimension of the histone code: when lactate exceeds a critical threshold, lactylation at the H3K18 locus (H3K18la) replaces acetylation at the H3K18 locus (H3K18ac), thereby inhibiting the recruitment of bromodomain-containing protein 4 (BRD4) and subsequent oncogene transcription (50). Lactyltransferases catalyze the covalent conjugation of lactyl groups to lysine residues, while delactylases mediate the reversible demodification process (48, 51). The initiation of histone lactylation depends on the activity of lactyltransferases, among which proteins of the p300/CBP family are key executors. As dual-functional enzymes with both acetyltransferase and lactyltransferase activities, p300 can utilize lactyl coenzyme A (lactyl-CoA) as a substrate to transfer lactyl groups to histone lysine residues (52). In HEK293T cells models, p300 overexpression significantly elevates histone lactylation levels, whereas shRNA-mediated p300 knockdown reduces H3K18la to 35% of controls (53). Zhang’s team further demonstrated in bone marrow-derived macrophages (BMDMs) that p300 deficiency abolishes lactate-induced H3K18la modification and suppresses pro-inflammatory cytokine (e.g., IL-1β) expression (8, 35). This catalytic specificity is associated with the bromodomain of p300, which preferentially recognizes lactyl groups bearing hydroxyl groups (54). Zhao (55) et al. identified class I histone deacetylases (HDAC1-3) as efficient erasers for both L/D-lactyllysine. Cellular studies revealed HDAC1 specifically regulates nuclear histone lactylation modifications (such as H4K12la), while HDAC3 is responsible for eliminating non-histone lactylation in the cytoplasm. Moreover, HDAC3 exhibits higher selectivity for lactylation modifications over acetylation modifications, as its catalytic domain can recognize the spatial conformation of lactyl-lysine and preferentially remove lactyl groups (56). Lactylation modification exhibits multi-compartmental distribution and broad target specificity: although initially discovered in histones, it is widely present in the nucleus, Lysosome (57), mitochondria (58), endoplasmic reticulum (59), and cytoskeleton (60, 61) (**Table 1**), and can modify not only histones but also non-histone proteins. In 2020, Gao et al. (62) discovered through their research that lactylation modification can occur on nuclear histones, cytoplasmic kinases, and mitochondrial enzyme complexes. For example, lactylation at the K147 site of Aldolase A (ALDOA) occurs most frequently and negatively regulates glycolysis formation through feedback inhibition (49). Additionally, lactate inhibits the Warburg effect by activating Pyruvate Kinase M2 (PKM2). Lactylation at the K62 site activates enzyme activity via an allosteric effect, driving the conversion of macrophages from a pro-inflammatory phenotype (M1) to a repair phenotype (M2), thus promoting IL-10-mediated inflammation resolution and wound healing (63).

**Table 1:** Relationship between major modification targets and disease in different cellular compartments.

Cellular compartment	Major modification targets	Biological functions	Disease association
Cell Nucleus	H3K18la/H4K12la	Chromatin remodeling/Gene transcription regulation	Colorectal Cancer (CRC)/Inflammatory Bowel Disease (IBD)
Lysosome	TFEB-K91la	Enhance lysosomal activity	Metabolic adaptation and proliferation of tumor cells
Mitochondria	H3K18la/PTMA	Inhibition of oxidative phosphorylation	Tumor immune evasion
Endoplasmic Reticulum	H3K18la/GP73	Promote angiogenesis	Hepatocellular carcinoma
Cytoskeleton	S100A11、IFI16、HSDL2	Immune cell migration and polarization/Inflammatory cytokine secretion	Ulcerative colitis
	α-tubulin-k40la	Enhance neuronal axon regeneration ability/Regulate neurite branching formation	Neurodegenerative diseases/Neuronal developmental abnormalities

In the nuclear compartment, the main modification targets are H3K18la/H4K12la, which are involved in chromatin remodeling and gene transcription regulation. Dysfunction of these targets is associated with the development of colorectal cancer and inflammatory bowel disease. In the lysosomal compartment, TFEB-K91la serves as the primary modification target. By enhancing lysosomal activity, it promotes metabolic adaptation and proliferation of tumor cells. In the mitochondrial compartment, modifications of H3K18la and PTMA inhibit oxidative phosphorylation, thereby leading to tumor immune escape and affecting the tumor microenvironment. In the endoplasmic reticulum compartment, H3K18la modification and the GP73 target promote angiogenesis, a process closely linked to the progression of hepatocellular carcinoma. In the cytoskeletal compartment, modification targets such as S100A11, IFI16, HSDL2, and α-tubulin-K40la regulate immune cell migration and polarization, cytokine secretion, and enhancement of neuronal axon regeneration capacity, respectively. Abnormalities in these targets are associated with diseases such as ulcerative colitis and neurodegenerative disorders (57–60).

## 3 Macrophage heterogeneity and activation regulatory networks

Macrophages, as innate immune cells with phenotypic plasticity and functional heterogeneity, play pivotal roles in maintaining tissue homeostasis and immune regulation. Based on developmental origin and functional states, tissue macrophages can be categorized into: ① tissue-resident macrophages (TRMs) of embryonic origin with self-renewal capacity; ② monocyte-derived macrophages (MDMs) differentiated from infiltrating circulating monocytes (64, 65). Macrophage activation refers to the dynamic phenotypic reprogramming in response to microenvironmental stimuli, such as metabolic remodeling (e.g., lactylation) and epigenetic modifications, whereas macrophage polarization refers to the activation state at a specific point in time that determines their functional phenotype (66), while macrophage polarization refers to the activation state of macrophages at a single point in time (67). In the *in vitro* characterization of macrophages, they can be distinguished into the classical activated M1 phenotype and the alternatively activated M2 phenotype based on surface receptor expression, secretion profiles, and functional activity (68, 69). Of course, some scholars believe that there are more than two phenotypes for macrophages based on their activation states. Therefore, the classification of macrophage biological behaviors remains an area that requires further research (70).

Classically activated M1 macrophages refer to those activated by IFN-γ secreted by TH1 cells and LPS (which induces TNF-α) stimulation. Alternatively activated M2 macrophages refer to those activated by IL-4 and IL-13 secreted by TH2 cells following *in vitro* stimulation (67, 71). M1 macrophages are believed to be pro-inflammatory (65).They can secrete high levels of pro-inflammatory cytokines (such as TNF-α, IL-1, IL-6, IL-23, etc.), enhance the microbicidal activity, and play an important role in anti-tumor immunity. M1 macrophages increase their cytotoxic activity by producing substances like superoxide anion, oxygen radicals, and nitrogen radicals, thereby promoting the inflammatory response. However, prolonged M1 activation can lead to tissue damage. In contrast, M2 macrophages secrete a variety of anti-inflammatory factors (e.g., IL-10, mannose receptor C-type 1), inhibit the levels of pro-inflammatory cytokines, promote the resolution of inflammation, and exert immunosuppressive effects. They play a role in preventing excessive inflammation and promoting tissue repair (68, 72–74).

In addition, M2 macrophages activate TGF-β by promoting the Th2 response, further facilitating fibrosis, which is closely associated with tissue remodeling (75). Persistent M2 phenotype can suppress the immune system, potentially increasing the risk of secondary infections or tumor development (76, 77). It is important to note that M2 macrophages can be further classified into subgroups based on the different stimuli they receive and the transcriptional changes that occur. Notably, according to the stimuli received and the transcriptional changes that occur, M2-type macrophages can be further divided into multiple subsets, including M2a, M2b, M2c, and M2d, with each subset playing distinct roles in immune responses and tissue repair processes (78). Currently, the most widely studied is the M2a macrophage subgroup, which has the functions of sensing and clearing pathogens and tissue remodeling (79). Macrophages can be polarized into the M2a phenotype by IL-3/IL-4 cytokines. The binding of pathogen-associated receptors to PRR triggers activation of clearance activity and activates downstream signaling cascades, secreting IL-10 to inhibit inflammatory responses (80, 81). Fibrosis is considered a potential biomarker of M2 macrophages. M2a expresses high levels of Fibronectin, and the produced chitinase-like substances play important roles in tissue reorganization (65, 82). In addition, M2a macrophages can assist tumor cells in growth through the IL-4/STAT6-mediated pathway, and IL-4 released by tumor cells and M2a macrophages further promotes the polarization of M2 macrophages into M2a, thus forming a positive feedback pathway (83). M2b macrophages, characterized by both immunomodulatory and pro-inflammatory properties, are also referred to as regulatory macrophages. These cells are induced by the classical M2b inducers lipopolysaccharide (LPS) plus immune complexes (ICs). Unlike other M2 subsets, M2b macrophages express Fcγ receptors (FcγR), which drive the secretion of high levels of the anti-inflammatory cytokine IL-10 and low levels of IL-12. They predominantly skew Th1 cell responses toward Th2 cell responses through IL-4 secretion. Polarization of macrophages toward the M2b phenotype requires two stimuli and involves multiple signal transduction events mediated by NF-κB, PI3K/Akt, IRF, and MAPK pathways (79, 84). In tumor progression, M2b macrophages gradually occupy the M1 cell population through the CC L1/CCR8 axis, thereby forming an immunosuppressive microenvironment (85).M2c macrophages, also known as acquired inactivated macrophages (86), are macrophages stimulated by IL-10, TGF-β, or glucocorticoids, characterized by secreting pro-inflammatory cytokines IL-10 and TGF-β, as well as chemokines CCL16, CCL18, and CXCL13 (79). In terms of L-arginine metabolism, M2c macrophages share the same metabolic state as M2a and produce Arg1, participating in fibrotic repair and progression, as well as wound healing (84, 87). M2d is an M2 subgroup of macrophages polarized by IL-6 and LIF, also exhibiting typical cytokine production characteristics of M2 subgroups (IL-10 ^high^, IL-12 ^low^) (88, 89). During M2d polarization, IL-6 induces M2d macrophage differentiation by activating JAK-STAT3-mediated cell signal transduction. In this process, macrophages consume M-CSF in an autocrine manner, and IL-6 and LIF play important roles in promoting this M-CSF consumption (88, 89). Additionally, M2d-polarized macrophages stimulated by adenosine, IL-6 and tumor cells secrete proteases (such as MMP-2), cytokines (such as VEGF) and anti-inflammatory factors (such as TGF-β and IL-10) to promote angiogenesis and tumor immunosuppression (90).

**Figure 2:**
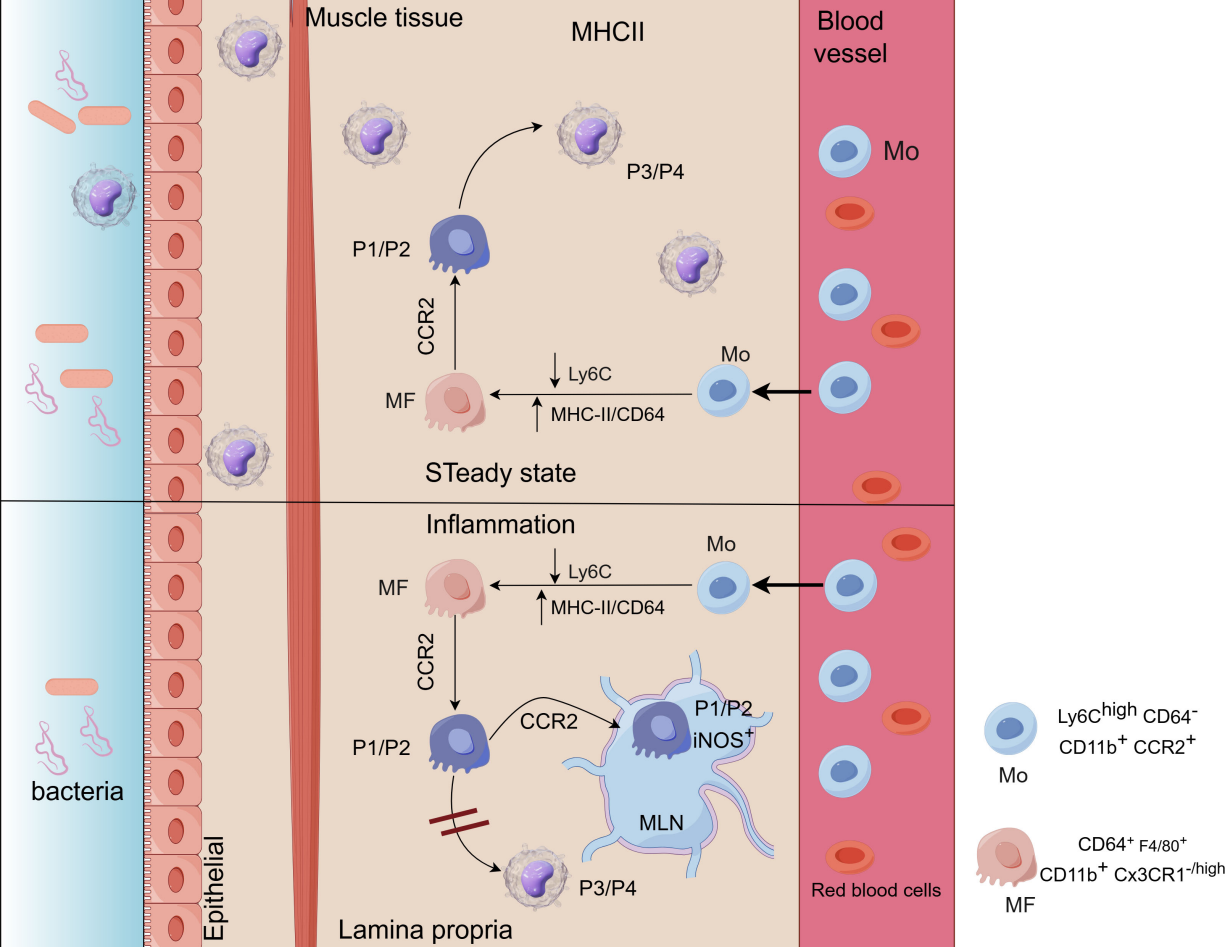
(Image 2 By Figdraw) Ly6C^hi^ blood monocytes (Mo) enter the intestinal lamina propria (LP) where they downregulate Ly6C and acquire the expression of MHC-II and CD64, becoming macrophages (MF). Up: under steady state conditions, Mo enter to the LP in a CCR2-dependent manner. They first go through P1/P2 stages (CD64^low/+^F4/80^neg^CX3CR1^−/int^) to finally become P3/P4 tolerogenic IL-10-producing monocytes (MF) (CD64^+^F4/80^+^CX3CR1^high^). Down: during inflammation MF differentiation is blunted, reaching only the P2-MF stage and becoming pro-inflammatory iNOS^+^ TNFα^+^ MF (92).

Macrophages in the gastrointestinal tract are classified into three subtypes based on their function and location: Subtype I (monocyte-derived mature macrophages), Subtype II (monocyte-derived inflammatory macrophages), and Subtype III (self-maintaining macrophages). These subtypes are involved in maintaining mucosal homeostasis, mediating acute inflammation, and regulating intestinal motility (91). Subtypes I and II of gastrointestinal macrophages are primarily located in the lamina propria. They are replenished by monocytes from the bloodstream and undergo a cascade of differentiation from P1 (newly recruited monocytes) to P4 (fully differentiated resident macrophages) (**Figure 2**). This differentiation process, which involves sequential stages from P1 to P4, is referred to as the monocyte waterfall (68, 93). Circulating Ly6ChiCCR2+ monocytes (P1) migrate to lamina propria via CX3CR1, sequentially acquire MHCII expression (P2), downregulate Ly6C (P3), and differentiate into mature CX3CR1^high^CD64^+^ macrophages (P4) over 7 days (91, 93–95). Tamoutounour et al. reported that macrophage differentiation is attenuated during inflammation, with macrophages in the lamina propria and lymphatic vessels only reaching the P2 stage (96). P2-stage macrophages express inducible nitric oxide synthase (iNOS) and secrete pro-inflammatory cytokines. However, the lack of CX3CR1 expression hinders the generation of fully tolerogenic IL-10-producing macrophages, leading to a predominantly pro-inflammatory phenotype (97). Studies have shown that mice deficient in CX3CR1+ cells are more susceptible to intestinal inflammation and more prone to developing intestinal inflammatory diseases (98). Subtype III is specifically found in the lamina propria and the outer layer of the muscularis in the gut. It is mainly composed of tissue-resident self-renewing macrophages, with additional replenishment from circulating monocyte-derived macrophages. Studies have shown that subtype III macrophages interact with the enteric nervous system (ENS), regulating intestinal motility and secretion. When subtype III cells are depleted, it can lead to a range of issues such as damage to the submucosal vascular network and weakened intestinal motility (78, 79, 99).

In colorectal cancer (CRC) progression, tumor-associated macrophages (TAMs) undergo phenotypic remodeling into M2-like immunosuppressive subsets, contributing to immune evasion and promoting tumor growth. Single-cell transcriptomics revealed significant upregulation of the immune checkpoint molecule CD155 in TAMs. This upregulation promotes IL-10 and TGF-β secretion through TIGIT-CD155 signaling, while suppressing IL-12p70 production. This creates a pro-tumorigenic microenvironment (100–102). Xu et al. (2021) first demonstrated that *Fusobacterium* nucleatum activates TLR4/NF-κB/miR-1322 signaling cascade to upregulate CCL20, driving monocyte differentiation into CCR6+ TAMs, which subsequently induce M2 polarization through IL-6/STAT3 pathway, upregulating Arg1 levels and down regulating iNOS levels, ultimately enhancing CRC liver metastasis (102, 103).

In gastric cancer (GC) microenvironments, M2 TAMs suppress CD8+ T cell cytotoxicityand NK cell IFN-γ secretion via PD-L1/IDO dual signaling. Clinical specimen analysis revealed positive correlation between TAM density and VEGF-C expression, promoting lymphangiogenesis (104, 105). Conversely, M1 macrophages induce GC cell ferroptosis through iNOS-dependent ROS production, while anti-CSF-1R antibody treatment significantly reduces TAM infiltration and reverses M2/M1 ratio (105).

According to statistics (106), more than half of patients with Crohn’s disease experience complications related to intestinal fibrosis. During Crohn’s disease (CD)-associated intestinal fibrogenesis, adherent-invasive *E. coli* (AIEC) activates TLR4/MYD88 signaling in intestinal epithelial cells, causing aberrant let-7b downregulation and subsequent loss of TGF-βR2 inhibition, driving macrophage transition towards profibrotic phenotypes. Animal models confirm that let-7b mimic administration reduces collagen deposition (106–109).

## 4 Metabolic reprogramming in macrophage polarization

The macrophage polarization process is accompanied by significant reprogramming of glucose metabolism and plays an important role in this process, characterized by dynamic shifts in energy metabolism pathways between M1 and M2 phenotypes. Emerging evidence suggests that metabolic reprogramming not only supplies energy and biosynthetic precursors but also determines cellular functional phenotypes through metabolism-epigenetics crosstalk. M1 macrophages rely predominantly on glycolysis and the pentose phosphate pathway (PPP), characterized by upregulated activities of HK2 and PFKFB3. Oxidative phosphorylation (OXPHOS) is suppressed via HIF-1α-mediated activation of PDK1, thereby disrupting the tricarboxylic acid (TCA) cycle. OXPHOS is suppressed via HIF-1α-mediated PDK1 activation, disrupting TCA cycle (110, 111). M2 macrophages exhibit enhanced OXPHOS and promotes fatty acid β-oxidation (FAO) via CPT1A, generating 2.5-fold more acetyl-CoA to support anti-inflammatory cytokine secretion (110, 112). HIF-1α is markedly upregulated in LPS-activated M1 macrophages, which binds promoters of glycolytic genes (e.g., GLUT1, LDHA) to enhance glycolysis and induces pro-inflammatory cytokines (IL-1β, TNF-α) (110). The pyruvate kinase M2 (PKM2) dimer forms a complex with HIF-1α, translocates to the nucleus to inhibit STAT1 phosphorylation, downregulating M1 markers (iNOS, IL-6) while promoting M2 signature gene (Arg1, IL-10) transcription (113, 114). In M1 macrophages, glycolytic activity is significantly enhanced, and ATP generation proceeds rapidly through the glycolysis pathway to provide sufficient energy for their antimicrobial and pro-inflammatory functions. This metabolic pattern is similar to the Warburg effect in tumor cells, where glycolysis is favored for rapid energy production despite adequate oxygen supply (110). The rapid progression of glycolysis leads to the accumulation of lactate, which not only provides energy for the cells but is also exported extracellularly via MCT4, altering the extracellular pH. This, in turn, inhibits T cell proliferation. At this stage, lactate can shuttle between cells and cellular compartments, serving as a metabolic substrate to provide energy (113). Polarization drives divergent arginine metabolism pathways: M1 macrophages express iNOS, while M2 macrophages express Arg1. Arginine is converted by iNOS and Arg1 into NO (nitric oxide), citrulline, ornithine, and urea, respectively (113).

## 5 Molecular mechanisms of lactylation in macrophage activation

**Figure 3:**
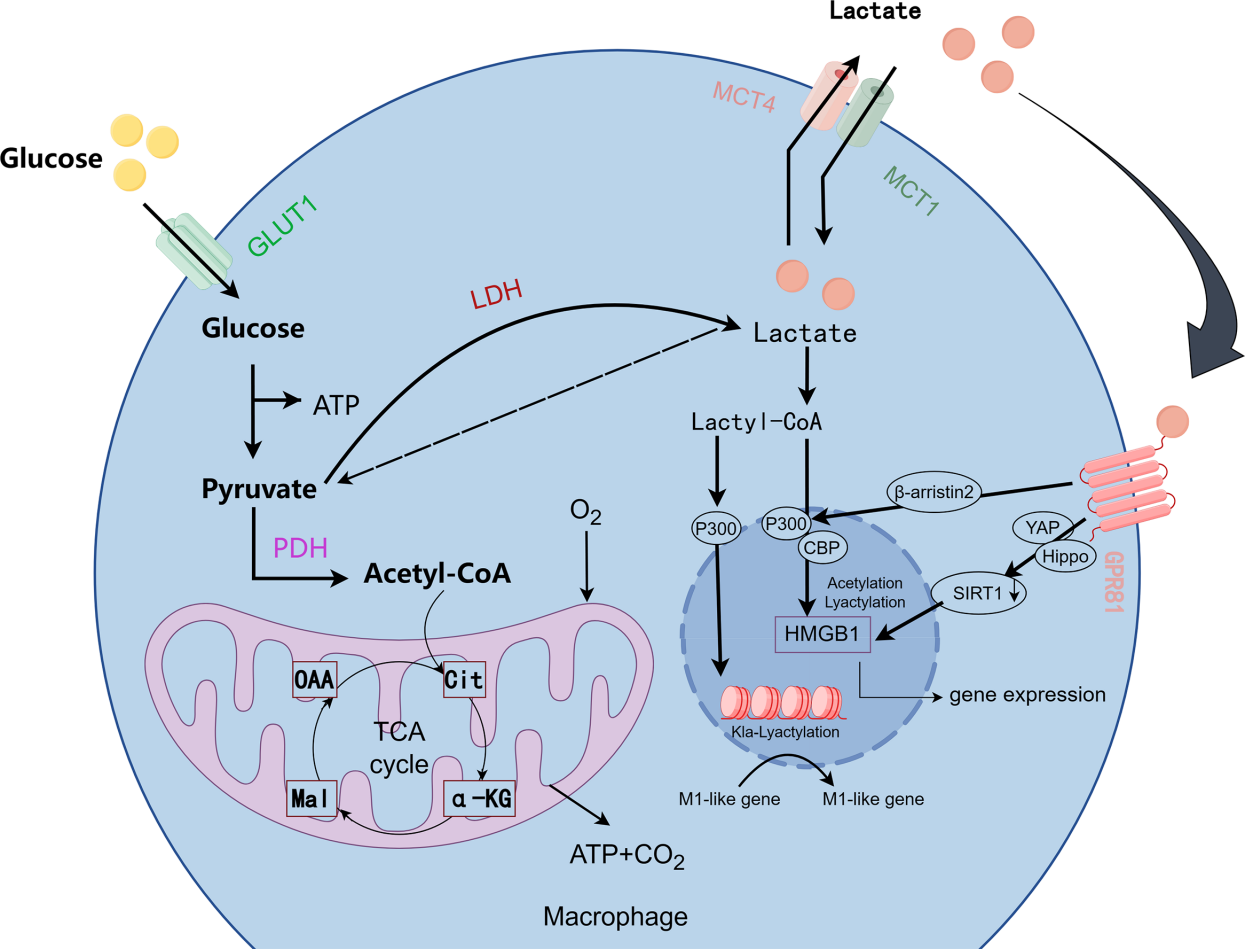
Metabolic pathways and lactylation in macrophages. (Image 3 by Figdraw) In the metabolic process of macrophages, glucose is converted into pyruvate, which then enters the mitochondria to generate ATP, or, under hypoxic conditions, is converted into lactate via lactate dehydrogenase (LDH). Lactate is exported out of the cell through the MCT1 and MCT4 transporters and can be converted into lactyl-CoA. Lactyl-CoA mediates the lactylation of proteins, including HMGB1, which regulates gene expression associated with M1 macrophage activation. Additionally, key signaling molecules such as GPR81, YAP, Hippo, and SIRT1 are involved in regulating these metabolic and immune responses.

Unlike histone methylation and acetylation, lactate, as a key metabolic regulatory molecule, regulates macrophage M1 phenotype polarization and the transition from M1 to M2 phenotypes through histone lactonylation modification and synergistic interaction with multiple signaling pathways (**Figure 3**) (115, 116).The “lactate clock” model proposed by Zhang et al. Zhang et al. proposed the “lactate clock” model, showing that M1-type macrophages accumulate lactate via the Warburg effect in the early phase of inflammation; subsequently, both extracellular lactate taken up by MCTs and enzymatically converted lactate-CoA can be enzymatically reacted to generate lactoyl-CoA, which can inhibit the expression of M1-related genes (e.g., TNF-α) and activate the repair program by mediating histone lactonylation via the p300/p53 complex (8, 113). This shifts macrophages from the P2 phase to the P3/P4 phase, enhances CX3CR1 expression, and reduces the secretion of inflammatory molecules, as well as the polarization of M2 macrophages. Zhang et al. (117) In pathological states such as spinal cord injury (SCI), microglia (brain-resident macrophages) have reduced CX3CR1 gene expression, decreased glycolysis, and reduced lactate production, leading to down-regulation of lactylation-related genes (e.g., Fabp5, Lgals1) expression, which results in an M1-like pro-inflammatory phenotype. While exogenous supplementation of lactate can inhibit the expression of pro-inflammatory genes (e.g., IL-1β, iNOS) in microglia through lactoylation modification, prompting the expression of CX1CR1 gene, and at the same time activate anti-inflammatory repair genes (e.g., Arg-1, CD206), promoting their polarization to M2 type (anti-inflammatory repair phenotype), thus reducing inflammation and improving the recovery of neurological function (117). In addition, related studies have found that CX3CL1 expression levels are gradually upregulated with increasing lactate concentration in gastric cancer cell lines and colon cancer cell lines (118). It is specifically enriched in the promoter region of repair genes during the late inflammatory phase, and promotes IL-10 secretion by inhibiting NF-κB phosphorylation, while activating the STAT6-driven transcription of the M2-type marker arginase-1, thereby attenuating the onset of inflammation (8, 115, 119). IL-4-induced M2-type polarization utilizes LDH1 to convert lactate into pyruvate, generating acetyl-coenzyme A, driving the histone acetylation-dependent gene expression (120). Lactate promotes vascular endothelial growth factor (VEGF) secretion by activating the mTORC1 signaling pathway and inhibiting ATP6V0d2-mediated degradation of HIF-2α lysosomes (121); lactate activates the mTORC2-AKT and ERK signaling pathways in tumor-associated macrophages (TAMs) leading to the up-regulation of PD-L1 expression (122); and lactate induces the expression of HF2α in tumor-associated macrophages (TAMs) through MCT1 endocytosis. MCT1 endocytosis induces K28 lactonylation of HMGB1 and inhibits SIRT1 deacetylase activity, thereby enhancing the transcription of DNA repair genes (123); other studies have found that lactic acid modifies lipopolysaccharides (LPS) in a time-dependent manner to activate macrophage histones and promote gene expression in M2-like macrophages, but interestingly, signature genes of traditional M2-type macrophages (However, it is interesting to note that the signature genes of traditional M2 type macrophages (e.g., Mrc1, Fn1, Retnla, etc.) are not significantly up-regulated, or may even be down-regulated, by LPS stimulation, which induces IL-6 secretion through the MyD88-dependent signaling pathway. IL-6 activates JAK2-STAT3 signaling through an autocrine/paracrine mode, which in turn promotes the expression of Arg1 and regulates arginine metabolism, resulting in macrophage polarization to the M2 phenotype and the process is dominated by glycolysis, and this is a major factor in the development of the M2 phenotype. This process is a specific glycolysis-dominated phenomenon independent of typical M2 polarization (124, 125). In sepsis models, lactate is transported from the extracellular space into macrophages via MCT1, which regulates the acetylation level of HMGB1 by inhibiting β-arrestin2-mediated nuclear translocation of p300/CBP acetyltransferase via the Hippo/YAP signaling axis (8, 24). Lactoylation of methyl-CpG-binding protein 2 (MeCP2 K271) leads to increased chromatin accessibility and transcriptional repression of RUNX1 in cellular models, promotes pro-repair M2 macrophage polarization, and maintains atherosclerotic plate stabilization (126). BCAP articulinic protein induces lactoylation of H4K12 via MyD88-IRAK4 signaling to activate ALOX15-mediated inflammatory reduction (127); furthermore, lactoylation activates DRP1, which induces mitochondrial fission to drive anti-inflammatory conversion (128). A study in traditional Chinese medicine found that Ge Gen Baicalin Lian Tang (GQD) inhibited HDAC3 and reduced lactoylation levels to alleviate DSS-induced colitis (129). Low pH in TME can independently alter macrophage phenotype and function. Lactate can promote lactoylation of lysine 18th position of histone H3 and activate CCL18 expression through macrophage Gpr132-mediated signaling pathway, while inducing polarization of M2 phenotype, which in turn promotes tumor proliferation and metastasis (8, 130). In addition, under hypoxic conditions, tumor necrosis factor superfamily member 9 (TNFSF9) expression is upregulated through a histone lactoylation-dependent mechanism, inducing polarization of M2-type macrophages and leading to malignant progression of tumor cells (131).

In the tumor microenvironment, lactate suppresses anti-tumor immune responses through negative feedback regulation of innate and adaptive tumor-infiltrating immune cells, and both the acidity of the TME and the increase in lactate affect macrophage polarization (132, 133). In glycolytic tumors, lactate concentrations can be elevated up to 40 mmol/g (134). Lactate produced by the Warburg effect in cancer cells becomes a key signal in the TME to induce M2 macrophage polarization (135). In normal tissues, pH_i_ (intracellular pH) and pH_e_ (extracellular pH) are around 7.2-7.4, compared to which tumors are able to maintain pH_i_ around 7.4 and pH_e_ down to around 6.5 (136). At pH 6.8, macrophage acidosis decreases the gene expression of pro-inflammatory markers Nos2, Ccl2 and Il-6 in IFN-γ/LPS-polarized macrophages (M1), while increasing the expression of anti-inflammatory markers Cd206, Arg1 and Reltna, as well as angiogenesis-related genes in IL-4-polarized macrophages (M2) (133). Moreover, the expression of CD206 and Arg1 in tumor-associated macrophages (TAM) was significantly reduced at physiological level of PH=7.4. The phosphorylation of AKT/ERK (mTOR downstream target) in macrophages was found to be enhanced in a lactate concentration-dependent manner; low concentrations of lactate (0–2 mmol/L) did not stimulate AKT-ERK well, while high concentrations of lactate (5–20 mmol/L) significantly activated the AKT-ERK signaling pathway; oxalate, a lactate inhibitor, reduced the concentration of LA in conditioned medium (CM) and counteracted high concentrations of LA. concentration in conditioned medium (CM) and counteracted the stimulatory effect of high LA concentration on this signaling pathway (137).

In pancreatic cancer, tumor cells were able to upregulate the lactate/METTL3/OAS3 (2,5’-oligoadenylate synthase 3) axis promoting M2d polarization (138). Moreover, in the context of lupus erythematosus, when macrophages are polarized to the M2b phenotype, notable alterations in macrophage glucose metabolism transpire, with enhanced glycolysis and the conversion of pyruvate to lactate by lactate dehydrogenase, leading to elevated lactate levels both intracellularly and extracellularly (139). Although the above shows that lactate produces some effects in M2b and M2d subtype polarization, the mechanism of lactate modification on the polarization of each subtype of M2 has yet to be thoroughly investigated. It is important to explore the mechanistic relationship between lactate modification on M2 macrophage subtypes for future target therapy of clinical diseases.

Triplex motif-containing protein 29 (TRIM29), a member of the TRIM family, is abundantly present in macrophages (140), mediating DNA binding, protein-protein interactions, and ubiquitin ligases, and is expressed in cancer, diabetic nephropathy, and immune-related disorders (141). PERK is a key metabolic hub for macrophage immune-suppressant function, and deletion of PERK signaling prevents mitochondrial respiration and lipid oxidation in M2 macrophages, impeding M2 macrophage polarization and promoting macrophage immunosuppression (142). Both play a role in inflammatory diseases of the gastrointestinal tract. One study (143) found that TRIM29 can control intestinal RNA virus-induced intestinal inflammation by targeting the NLRP6 and NLRP9b signaling pathways, while TRIM29 can also regulate PERK (144), which drives glucose metabolism and promotes macrophage immune-suppressing activity through histone lactylation (145), and thus regulates gastrointestinal inflammation. Thus, TRIM29 could alleviate gastrointestinal diseases by modulating inflammasome activation and lactylation-mediated PERK-ER stress immunosuppression. Su et al. found that G6PT-deficient macrophages induced lactate accumulation and reduced the activation of NLRP3 inflammatory vesicles through the lactylation-ALKBH5-m6A-NLRP3 pathway an enhancement of ALKBH5 expression and alleviate IBD (146, 147). In addition, in characteristic dermatitis (AD) studies, UV-treated riboflavin was found to inhibit the activation of NLRP3 inflammatory vesicles in macrophages by inhibiting the H3K9 lactation of NLRP3 and ASC, leading to a reduction in IL-1β secretion and M1 macrophage polarization, as well as a reduction in TSLP secretion by keratin-forming cells to attenuate AD progression (148).

## 6 Pathological mechanisms of lactylation-regulated macrophage activation in gastrointestinal diseases

Lactylation plays a crucial role in regulating macrophage activation and polarization, particularly in gastrointestinal diseases, where metabolic changes in lactate are closely linked to macrophage function. Gastrointestinal diseases such as ulcerative colitis (UC), Crohn’s disease (CD), and gastrointestinal cancers are strongly associated with immune cell dysfunction and inflammatory responses. Lactylation, by modulating macrophage immune responses, provides a new direction for research into the treatment of these diseases (**Table 2**).

**Table 2:** Specific mechanisms of action and potential targets of lactation in various diseases.

Classification of diseases	The role oflactation	Potential targets
inflammatory boweldisease (lBD)	Lactate inhibits the pro-inflammatoryactivity of Mi macrophages byinteracting with GPR81 and promotesthe reparative phenotypictransformation of M2 type, which helpsalleviate the inflammatory response.	GPR81.lactate/LDHi pathway
Postoperative intestinalobstruction (PIO)	An increase in lactate can lead tohistone lactation,which promotesCXCL1 secretion through the AKT.mTOR signaling pathway,increasingthe risk of inflammation and intestinalobstruction.	including VCAMi andAKTmTOR signaling pathways
Gastrointestinal tumors	Lactate accumulation in the tumormicroenvironment promotes thetransformation of macrophages to M2type, enhancing the growth andmetastasis ability of tumor cells.	AKT-mTOR signaling pathway, VCAM1

### 6.1 Inflammatory bowel disease

Inflammatory bowel disease (IBD), which encompasses ulcerative colitis (UC) and Crohn’s disease (CD), is an incurable chronic inflammatory gastrointestinal disorder characterized by chronic intestinal inflammation, immune dysregulation, and prominent metabolic-epigenetic crosstalk (149, 150). While causing damage to the intestine, it can also significantly impair the patient’s quality of life (151). Single-cell metabolomics reveals glycolytic reprogramming in intestinal macrophages of IBD patients (152). Studies have found that histone lactylation modifications enhance the expression of genes involved in inflammatory responses (153). H3K18 lactylation enhances YTHDF2/Kcnk6 complex stability, activating NLRP3 inflammasome (2.3-fold ASC oligomerization) and promoting IL-1β secretion (154). In ulcerative colitis, lactate in its ionic form downregulates cyclic AMP (cAMP) and protein kinase A (PKA) signaling through the GBR81 receptor, inhibiting the expression of the M1 marker iNOS. At the same time, it activates PPARγ to promote the transcription of M2-type Arg1, thereby alleviating the occurrence of inflammation (155). Lactate accumulation in macrophages lactylates the PKM2 K305 site to stabilize the tetrameric conformation, providing negative feedback inhibition of glycolytic flux and blocking M1 polarization (27, 63). Additionally, studies have found that TAK-242 inhibits the recruitment of MyD88 and TRIF to TLR4 by binding to the TIR domain of MyD88/TRIF, reducing NF-κB phosphorylation and MAPK activation. At the same time, it promotes the expression of anti-inflammatory genes mediated by H3K9la, facilitating tissue repair (153, 156, 157). It has been reported that the lactate content in the gastrointestinal tract of Crohn’s disease (CD) patients is significantly elevated (158). Macrophages exhibit high levels of lactylation, and the expression of SIRT1 is notably reduced, negatively correlating with the degree of oxidative phosphorylation. It is also negatively correlated with the infiltration of pro-inflammatory immune cells, such as Th17 cells, which are regulated by the monocarboxylate transporter encoded by SLC16A1 (24, 113). In a study by Sun et al. (159) on lactate-producing yeast in ulcerative colitis, it was found that lactate from the yeast upregulates NLRP3 transcription via MCT1, while inhibiting H3K9 acetylation and promoting H3K18 lactylation, thus alleviating DSS-induced colitis damage. Additionally, under the guidance of traditional Chinese medicine theory, Gegen Qinlian Decoction (GQD) is an effective prescription for treating ulcerative colitis. Xu et al. experimentally verified that GQD inhibits HDAC3 activity, reducing histone H3/H4 lactylation levels and reversing the imbalance between M1/M2 polarization, providing molecular evidence for the use of traditional Chinese medicine in treating IBD (129).

### 6.2 Lactylation regulatory mechanisms in postoperative ileus

Postoperative ileus (POI) is one of the common complications following gastrointestinal surgery. The occurrence of POI can affect gastrointestinal function and prolong postoperative recovery time for patients (160–162). According to a report by Grocott, 92% of general surgery patients experience gastrointestinal postoperative complications (163). Surgical trauma activates resident macrophages in the intestinal muscular layer, leading to the release of cytokines and chemokines, and the recruitment of leukocytes (160, 164). Among these, the M1 macrophages, in response to mechanical stimuli, release circulating chemokine ligand 1 (CXCL1) via the TLR4/MyD88 signaling axis. The increase in CXCL1 leads to increased intestinal tension, a reduction in contraction amplitude, and neutrophil infiltration (163). Postoperative inflammation enhances glycolysis, thereby increasing lactate production. Lactylation of H3K18, by enhancing the accessibility of the VCAM1 promoter, activates the AKT/mTOR signaling pathway, promoting the continuous secretion of CXCL1. This leads to the formation of a malignant cycle of inflammation and motility dysfunction (55, 165, 166). STAT3 K685 lactylation strengthens DNA binding, promoting IL-6 autocrine loops and enteric glia activation (125). Therefore, we can treat or prevent postoperative ileus by employing therapeutic strategies that target lactylation regulation:1. Inhibiting MCT1-mediated lactate influx2. Blocking HDAC3-dependent histone lactylation3. Modulating STAT3 lactylation.

### 6.3 Lactylation regulatory network in gastrointestinal malignancies

Gastrointestinal cancers, including colorectal cancer (CRC) and gastric cancer (GC), remain leading causes of global cancer-related mortality (167). Epigenetic alterations and metabolic reprogramming play pivotal roles in oncogenesis (168). The tumor microenvironment (TME), a heterogeneous ecosystem, harbors diverse tumor-associated macrophage (TAM) populations classically categorized into anti-tumor M1 and pro-tumorigenic M2 subtypes (169, 170). Emerging evidence reveals that pro-tumor TAMs exhibit cancer type-specific molecular signatures (171), with lactate gradients potentially driving their polarization through GPR81-mediated mTORC1 signaling (135). Lactate, a key glycolytic byproduct in cancer metabolism, functions as an epigenetic modulator via histone lysine residue lactylation, thereby influencing tumor progression (8, 22, 172, 173). Notably, crosstalk between lactylation and histone acetylation has been identified; for instance, H3K18 lactylation may competitively inhibit HDAC3 activity, amplifying H3K27 acetylation to synergistically activate oncogenic transcription (8).

In CRC, Li et al. demonstrated that Warburg effect-derived lactate promotes H3K18 lactylation, suppresses macrophage RARγ expression, disrupts TRAF6 interactions, elevates IL-6 levels, and activates STAT3 signaling, collectively driving colorectal tumorigenesis (124). Single-cell metabolomics further revealed that M2-TAM subsets preferentially uptake lactate via upregulated monocarboxylate transporter 1 (MCT1), relying on lactate dehydrogenase A (LDHA) to sustain their pro-tumor phenotype (174). Proprotein convertase subtilisin/kexin type 9 (PCSK9) enhances colon cancer progression by modulating epithelial-mesenchymal transition (EMT) and PI3K/AKT signaling while skewing macrophage polarization (175). Additionally, gut microbiota-derived lactate reprograms ATM glycolysis, induces RIG-I K852 lactylation to inhibit RIG-I-MAVS-NF-κB signaling, and synergizes with cathepsin K to establish an immunosuppressive niche favoring CRC metastasis (176, 177). It is worth noting that this microorganism-host metabolic interaction is cancer-type specific. For example, in ductal carcinoma of the breast, stellate cells, rather than microorganism-derived lactate, drive TAM immunosuppressive function through the CCL5-CCR5 signaling axis (178).

While RIG-I lactylation promotes M2-like polarization by rewiring macrophage metabolism and inflammatory pathways (176), paradoxical evidence suggests that localized lactate accumulation at high concentrations may transiently suppress tumor growth via caspase-1-mediated pyroptosis (179). Concurrently, diminished anti-tumor activity of regulatory T cells (Tregs) and CD8+ T cells further compromises immune surveillance. LDHA inhibitors (e.g., FX-11) reverse TAM polarization and synergize with PD-1 blockade, offering novel combinatorial strategies for gastrointestinal malignancies. In GC models, lactate-induced H3K18 lactylation upregulates VCAM1 transcription, activates AKT-mTOR signaling, enhances CXCL1 secretion, and expands GC-derived mesenchymal stem cells and M2 macrophages, collectively accelerating gastric cancer progression (55). Nevertheless, technical limitations persist, particularly the lack of spatial metabolomics integrated with single-cell transcriptomics to resolve TAM metabolic heterogeneity (180).

The role of lactate in different diseases and its potential therapeutic targets. In inflammatory bowel disease (IBD), lactate inhibits the pro-inflammatory activity of M1 macrophages by interacting with GPR81 and promotes the reparative transformation of M2 macrophages, thereby alleviating the inflammatory response (155). In postoperative intestinal obstruction (PIO), increased lactate leads to histone lactylation, which promotes CXCL1 secretion through the AKT-mTOR signaling pathway, increasing the risk of inflammation and obstruction (163). In gastrointestinal tumors, lactate accumulation in the tumor microenvironment promotes the transformation of macrophages to the M2 phenotype, enhancing tumor growth and metastasis. Potential therapeutic targets include GPR81, the lactate/LDH1 pathway, VCAM1, and the AKT-mTOR signaling pathway (55).

### 6.4 Bacterial and viral infectious gastrointestinal diseases

Unlike non-infectious gastrointestinal diseases, lactation modifications also play an important role in the pathogenesis of bacterial and viral infectious gastrointestinal diseases by controlling macrophage polarization. In recent years, it has been demonstrated that viruses and bacteria are capable of disrupting host metabolic homeostasis during the first phase of infection, specifically the “Warburg” effect (181).There seems to be a relationship between lactonization and bacterial metabolism. One study (182) found that lysine lactonization can regulate the metabolic pathways of Streptococcus mutans. And when we used some catalysts to promote lysine lactylation in E. coli (183), we found that E. coli enhanced the production of lactic acid, which mediates the polarization of M2 macrophages through inhibition of nuclear factor κB gene binding, and undergoes a pro-inflammatory response that leads to liver metastasis of colorectal cancer (176). Additional studies (184) have also found that protein lactylation modulates the intestinal microflora, leading to cancer cell migration and metastasis. Therefore, it has been investigated (159) that by modulating the intestinal microbiota, regulating macrophage polarization status and inhibiting the expression of pro-inflammatory cytokines, inhibiting the over-activation of NLRP3 inflammatory vesicles and the downstream caspase-1 pathway in macrophages, and, at the same time, promoting protein lactonization, attenuates inflammation in ulcerative colitis.There is an association between lactonization and the viral life cycle. A study (185–188) identified a correlation between lactation and the replication and reactivation of herpes simplex virus, severe fever with thrombocytopenia syndrome virus, and Kaposi’s sarcoma-associated herpesvirus, with many of these viruses increasing cellular lactate levels. It has also been found that viruses modulate protein lactylation, leading to disease progression (192). The two appear to be a reciprocal relationship. However, there are no studies that have found any relationship between lactic acidification and viruses associated with gastrointestinal diseases, which could be a direction for future research.

## 7 Conclusion

Since the groundbreaking discovery of histone lactylation by the Zhang (8) research team in 2019, this field has rapidly transitioned from foundational mechanistic exploration to translational therapeutic development. Emerging evidence has systematically elucidated the dual roles of lactate: functioning not only as a metabolic intermediate to facilitate energy supply but also as an epigenetic regulator that remodels the immune microenvironment through lactylation. As central effector cells of the innate immune system, macrophages exhibit spatiotemporally specific regulation of polarization states (M1/M2 dynamic equilibrium) during gastrointestinal pathogenesis, with lactylation acting as a pivotal mediator via metabolic-epigenetic crosstalk mechanisms. This review comprehensively discusses the impact of lactate-mediated lactylation on macrophage polarization in gastrointestinal diseases. Deciphering the mechanistic interplay between lactylation and macrophage polarization holds significant promise for developing innovative therapeutic strategies.

As a nascent research direction, several critical challenges remain unresolved:The precise regulatory mechanisms underlying lactylation-dependent modulation of macrophage polarization require further elucidation. The regulatory networks governing lactylation, particularly the identification of tissue-specific enzymatic systems (e.g., lactyltransferases and delactylases), remain incompletely characterized. The interplay between lactylation and other post-translational modifications (PTMs), such as acetylation and succinylation, demands systematic investigation to clarify their synergistic or antagonistic effects. Biomarker discovery based on lactylation signatures for disease staging and therapeutic monitoring represents an unmet need. Whether lactylation modifications could provide novel insights into the therapeutic mechanisms of traditional Chinese medicine (TCM) warrants interdisciplinary exploration.

In conclusion, advancing our understanding of lactylation-mediated macrophage polarization in gastrointestinal pathophysiology will accelerate the identification of druggable targets and the development of combinatorial therapeutic regimens, ultimately opening new frontiers for the treatment of gastrointestinal malignancies.
